# Primates in Burrows: A Cause for Concern? Observations From a One Health Perspective at Niokolo Koba National Park, Senegal

**DOI:** 10.1002/ece3.71062

**Published:** 2025-04-07

**Authors:** Cédric Vermeulen, Jérôme Vandebos, Daelemans Virginie, Simon Lhoest

**Affiliations:** ^1^ Forest is Life University of Liège, Gembloux Agro‐Bio Tech Gembloux Belgium

**Keywords:** burrow, camera trap, primate, zoonosis

## Abstract

Burrows are places where many species cross paths and potentially exchange diseases. Primates are rarely frequenting burrows. However, this brief descriptive communication shows that three species of primates in the Niokolo Koba National Park (Senegal) frequent the vicinity of burrows, with some individuals even entering them. In particular, these primates come potentially into contact with bats. We aim to draw the scientific community's attention to the fact that burrows serve as critical interaction points for various taxa, some of which are reputed to carry zoonoses. As such, these burrows should be considered as potential “One Health hotspots” to be monitored, especially when frequented by primates in contact with rangers or tourists.

## Graphical abstract

Burrows are places where many species cross paths and potentially exchange diseases. Primates are not known to frequent burrows. However, in the Niokolo Koba National Park (Senegal) primates entering in burrows and come potentially into contact with other species, including bats. From a One‐health perspective, we draw attention to the need to further document these inter‐specific interactions.

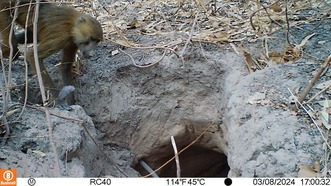

Fifty‐eight percent of terrestrial mammals use burrows. Some species dig their own burrows, while others only occupy preexisting burrows (Kinlaw 1999). Burrows play a vital role in the survival of wildlife in arid and semi‐arid environments. They provide shelter from predators and during bushfires and maintain a wildlife‐friendly microclimate with more stable temperatures and humidity than outside (Kinlaw 1999; Whittington‐Jones et al. 2011).

Several species can cohabit within the same burrow. For example, aardvark ( *Orycteropus afer* Pallas, 1766) burrows can be used by more than 20 species of mammals, birds, and reptiles in a year (Whittington‐Jones et al. 2011). Lehmann et al. (2020) showed in Gabon that giant pangolins (*Smutsia gigantea* Illiger, 1815) share burrows with African brush‐tailed porcupines ( *Atherurus africanus* Gray, 1842), white‐bellied pangolins (*Phataginus tricuspis* Rafinesque, 1821), small rodent species (order *Rodentia*), genets (*Genetta* spp. Cuvier, 1816), mongooses (e.g., *Atilax paludinosus* Cuvier, 1829), and microbats (suborder *Microchiroptera*). However, these interspecies cohabitations within burrows remain poorly documented. This proximity increases the probability of disease transmission from one species to another, especially when they are also hosting ticks capable of transmitting various pathogens. Burrows can, therefore, be potential sites for interspecies contamination (Lehmann et al. 2020). Some of these diseases, known as zoonoses, can be transmitted to humans. Indeed, most infectious agents impacting humans are zoonoses, and 70% of these have wild animals as hosts (Kumar et al. 2013). Moreover, three main orders (rodents, bats, and primates) are collectively involved as carriers of the majority (76%) of zoonotic viruses described to date (Johnson et al. 2020). It is now recognized that a collaborative, multisectoral, and transdisciplinary One Health approach (Narrod et al. 2012) including improved knowledge and monitoring of ecosystem health, is required to achieve optimal health outcomes for people, animals, and ecosystems. Burrows, as a potentially important sites for the exchange of zoonotic diseases between wild species, deserve to be actively monitored. This is particularly true in Senegal, where zoonoses such as rabies are considered significant health issues (Msolo et al. 2020; Faye et al. 2022). The Niokolo Koba National Park, with the most diverse terrestrial mammal community in the country (Gueye et al. 2022), is therefore a prime study site from this perspective, especially given that the tourist camps it hosts are frequented by primates in regular interaction with rangers and tourists (Mediannikov et al. 2020).

This preliminary descriptive study aims to document the use of burrows by different species (especially primates) in Niokolo Koba National Park around the Niokolodge (an eco‐tourism facility).

We characterized and monitored 92 burrows within a 7 km radius of the Niokolodge (42 burrows from February to May 2023 and 50 burrows from March to April 2024), targeting camera traps at 105 burrow entrances. Camera traps have already been used to monitor various species of mammals occupying burrows, including bats (Kondo 2018). This is a particularly effective tool for monitoring rare and elusive species. Bushnell Core DS‐4 K cameras were used to monitor burrows. Camera parameters were adjusted: image size was set to “medium” to avoid saturation of SD cards, the “fast motion” option was activated to maximize detections, and video duration was set at 15 s, with a trigger delay of around 0.5 s after motion detection (Fonteyn et al. 2022). The sensor was set to “High”, as recommended in the recommendations of the Bushnell user guide (2021), this setting being suitable for conditions where air temperature exceeds 40°C to improve detection capability. The videos captured by the camera traps were manually sorted using the Timelapse software (version 2.3.0.6). For each video, the following information was recorded: the species identified, based on “The Kingdon Field Guide to African Mammals” (Kingdom, 2013), the time of day (night/day), the animal interest in the burrow, and penetration of a number of individuals. Two events were considered independent if they were separated by an interval of at least 30 min.

The burrows observed were all dug by aardvarks and measured around 60 cm diagonally at the entrance. The number of entrances per burrow was highly variable, with 1–8 entrances observed. For each burrow, we oriented the camera to face the most used entrances (determined based on animal tracks and traces). The cameras were mounted on a stake 1 meter from the burrow entrance to observe the interior and were protected from the sun using a small handmade roof. Between February and April 2023 and March and April 2024, we monitored each burrow entrance for 10–20 days using a single camera trap. The total sampling effort was 1460 camera days.

We analyzed the species occurrences in and around each burrow. We observed three species of terrestrial primates (patas monkey *Cercopithecus patas* Schreber, 1774, vervet monkey *Cercopithecus sabaceus* Linnaeus, 1766, Guinea baboon *Papio papio* Desmarest, 1820), demonstrating genuine interest in burrows occupied mainly by bats and warthogs (Table 1).

**TABLE 1:** Summary of the number of detection events by species or species groups during monitoring of 42 burrows by camera traps from February to May 2023 and 50 burrows from March to April 2024, according to three classes.

Species	1	2	3	Total
**Patas monkey (*Cercopithecus patas* Schreber, 1774)**	12	6	2	20
**Vervet monkey (*Cercopithecus sabaceus* Linnaeus, 1766)**	11	2	2	15
**Guinea baboon (*Papio papio* Desmarest, 1820)**	36	4	5	45
Bats (*Chiroptera*)	109	362	1021	1492
Common warthog ( *Phacochoerus africanus* Gmelin, 1788)	19	40	176	235

*Note:* Class 1 (animal observed indifferent to the burrow entrance), class 2 (animal interested in the burrow entrance), and class 3 (animal entering or exiting the burrow entrance). Primate species are shown in bold.

All three primate species are interested in burrows. They either forage in the entrance (Class 2, Table 1) or even penetrate the interior (Class 3, Table 1). These findings corroborate the observations of Turner et al. (2019) with grivet ( *Chlorocebus aethiops* Linnaeus, 1758), which use aardvark burrows to protect themselves from the heat. Observations of primates around and within burrows at Niokolo Koba are proportionally much less frequent than other taxa (Table 1), but the observations made are not just random captures of individuals passing in front of the camera; primates show interest in the burrows, foraging around and at the entrances, and sometimes entering, likely in search of insects and arthropods taking refuge inside during the dry season (see videos 1, 2, 3 and 4 in Supporting Information).

**VIDEO 1:** Baboon_indifference. Video content can be viewed at https://onlinelibrary.wiley.com/doi/10.1002/ece3.71062

**VIDEO 2:** Baboon_in_burrow. Video content can be viewed at https://onlinelibrary.wiley.com/doi/10.1002/ece3.71062

**VIDEO 3:** Baboon‐foragins. Video content can be viewed at https://onlinelibrary.wiley.com/doi/10.1002/ece3.71062

**VIDEO 4:** Patas_interest. Video content can be viewed at https://onlinelibrary.wiley.com/doi/10.1002/ece3.71062

We also observed species co‐occurrences. A co‐occurrence is defined in this study as two species, each represented by at least one individual, entering the same entrance belonging to the same burrow during the observation period by cameras. We observed 13 burrows in which bats entered an entrance where the presence of baboons was recorded nearby (see for example videos 5 and 6 of baboons and chiropterans emerging from the same burrow 10 days apart). Bats roost indoors but enter and exit many times a night. Baboons and bats were recorded entering the same entrance in two burrows (Table 2). We also recorded several co‐occurrences between primates and other species, including two co‐occurrences of Guinea baboon *Papio papio* with the guinea fowl *Numidia meleagris*, one co‐occurrence of *Papio papio* with the common warthog *Phacochoerus africanus* , one co‐occurrence of *Papio papio* and the aardvark *Orycteropus afer* (see video 7, 8 and 9 where the warthog, aardvark and fowl emerges from the same entrance as the Baboons foraging in video 3 in Supporting Information), one co‐occurrence of *Papio papio* and stone partridge *Ptilopachus petrosus* , one co‐occurrence of the vervet monkey *Cercopithecus sabaeus* and *Numidia meleagris*, one between *Cercopithecus sabaeus* and the aardvark *Orycteropus afer* , and finally one co‐occurrence between *Cercopithecus patas* and *Numidia meleagris*. Even if primate penetration of burrows is not very frequent, the three species of primates in the park are confirmed to frequent burrows occupied by a wide variety of taxa.

**VIDEO 5:** Baboon_entry_sameChiro. Video content can be viewed at https://onlinelibrary.wiley.com/doi/10.1002/ece3.71062

**VIDEO 6:** Chiro_entry_sameBaboon. Video content can be viewed at https://onlinelibrary.wiley.com/doi/10.1002/ece3.71062

**TABLE 2:** Co‐occurrence between species of primates of Niokolo Koba National Park and other taxa during monitoring of 42 burrows by camera traps from February to May 2023 and 50 burrows from March to April 2024.

Species of primate	Co‐occurence	Nombre
Guinea baboon ( *Papio papio* Desmarest, 1820)	Helmeted guineafowl (*Numidia meleagris* Linnaeus, 1758)	2
	Aardvark ( *Orycteropus afer* Pallas, 1766)	1
	Bats (*Chiroptera*)	2
	Common warthog (*Phacocherus africanus* Gmelin, 1788)	1
	Stone partridge ( *Ptilopachus petrosus* Gmelin, 1789)	1
Vervet monkey (*Cercopithecus sabaceus* Linnaeus, 1766)	Aardvark ( *Orycteropus afer* Pallas, 1766)	1
	Helmeted guineafowl (*Numidia meleagris* Linnaeus, 1758)	1
Patas monkey (*Cercopithecus patas*, Schreber, 1774)	Helmeted guineafowl (*Numidia meleagris* Linnaeus, 1758)	1

**VIDEO 7:** Phaco_entry. Video content can be viewed at https://onlinelibrary.wiley.com/doi/10.1002/ece3.71062

**VIDEO 8:** Entry_aardvark. Video content can be viewed at https://onlinelibrary.wiley.com/doi/10.1002/ece3.71062

**VIDEO 9:** Entry_guinea_fowl. Video content can be viewed at https://onlinelibrary.wiley.com/doi/10.1002/ece3.71062

These interspecies burrow visitations can potentially lead to interspecies transmission of pathogens common to the species involved, as already demonstrated by Alexander et al. (2015) concerning pathogen transmission between primates and bats. Among these, the most concerning for humans in Senegal are likely *Cryptosporidium* (an infectious agent causing severe diarrhea and a significant health issue to people in developing countries and carried in particular by cats, dogs, and cattle) and the rabies virus (notably carried by bats and terrestrial carnivores), which are considered significant health concerns (Faye et al. 2022; Msolo et al. 2020; Sow et al. 2016). Bats are known to harbor and transmit the most documented zoonoses to humans (Johnson et al. 2020), and their sharing of burrows with primates is a source of concern, especially if we remember that transmissions between primates and bats have already led to epidemics of deadly viruses such as Ebola (Alexander et al. 2015). Moreover, we observe the presence of chiropterans *Nycteris macrotis* in burrows. This species could present zoonotic risks, as it was identified in Guinea by Lacroix et al. (2020) as a carrier of β‐coronavirus (β‐CoV) belonging to the MerbeCoV subgenus, which also includes other coronaviruses similar to MERS‐CoV‐like viruses capable of being transmitted between humans and animals. These findings justify specific monitoring, particularly on the outskirts of the park, where wildlife‐human interactions are most significant. From a One Health perspective, monitoring the interactions of vervet monkeys with other species is critical. These monkeys are both crop predators frequently observed in human‐impacted environments and animals that interact with tourists and guides in the camps of Niokolo Koba National Park (see Photo S1 and S2 in Supporting Information). For instance, Mediannikov et al. (2020) demonstrated the presence of *Treponema pallidum* subsp. *pertenue*, a bacterium belonging to the same group as those responsible for human venereal syphilis in green monkeys within tourist camps of Niokolo Koba Park. This strain is the agent of pian disease, which causes chronic skin infections in humans characterized by warty tumors on the skin that develop into ulcers.

From the burrow to the tourist camp, a whole range of species interact (Figure 1), interactions that need to be documented and understood for the sake of prevention.

**FIGURE 1:**
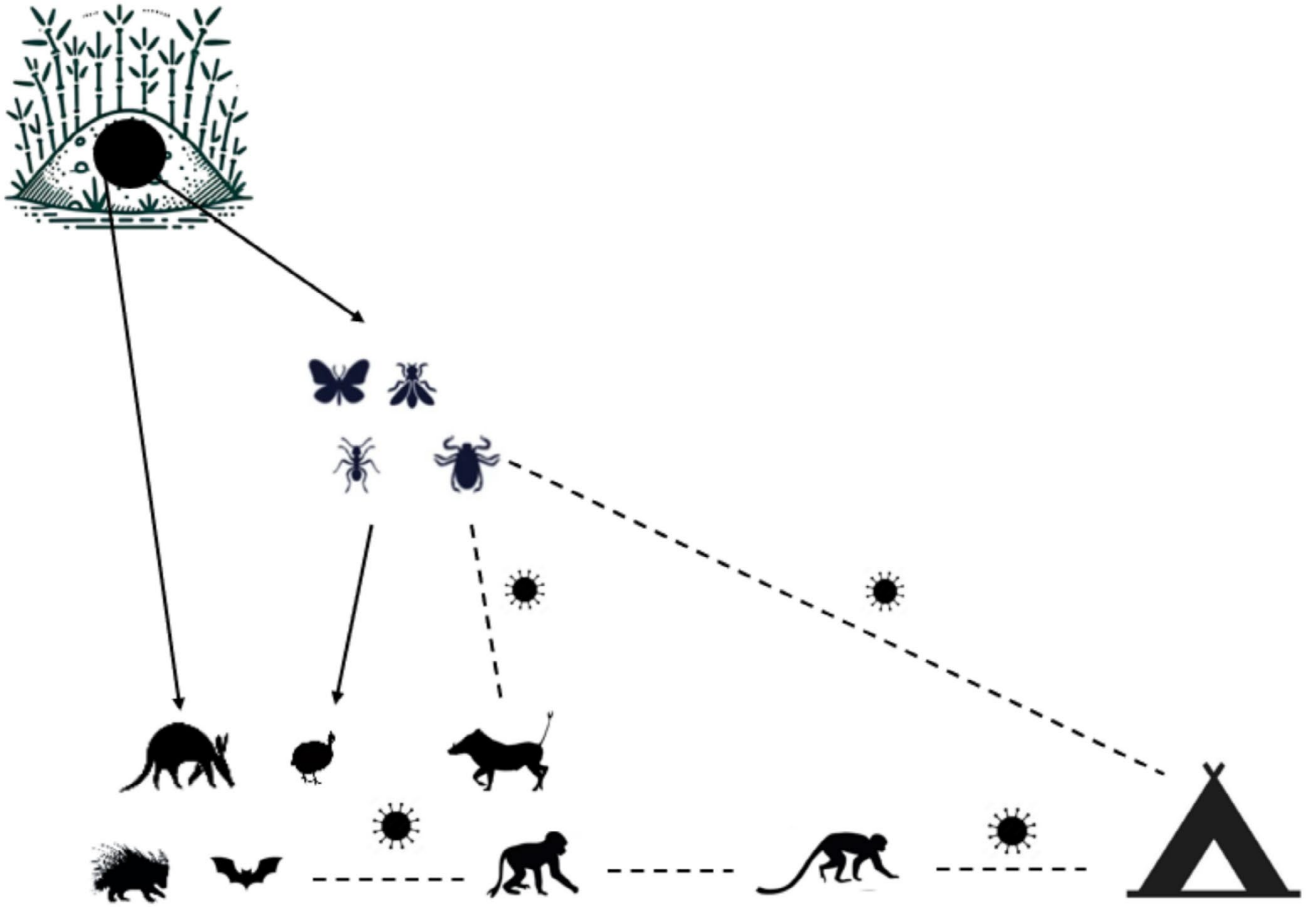
Zoonotic risks associated with burrows. Solid arrows indicate which species are attracted to burrows; dotted arrows indicate transmission risks. The figure illustrates the interactions between different animal species, insects, arthropods, and humans. In the upper left‐hand section of the diagram, burrows occupied by different mammal species can harbor a variety of insects and arthropods, including ticks such as *Rhipicephalus sanguineus* , which carry potentially zoonotic pathogens and can infect burrow fauna. The arrows in the diagram indicate the risk of transmission of pathogens and zoonoses. Among the mammals present in the burrows, chiropterans, such as *Nycteris macrotis* , can be carriers of high‐risk pathogens and potentially come into contact with primates such as *Chlorocebus sabaeus* , themselves interacting with humans in tourist camps.

The limitations of this preliminary study suggest future research directions: ensuring the identification of all bat species through passive acoustic methods, accounting for Senegal's marked seasonality, and identifying pathogens shared between primates and bats.

We assume that this first descriptive contribution does not provide conclusive evidence of pathogen or zoonosis transmission in or around burrows. However, we aim to draw the scientific community's attention to the fact that burrows serve as critical interaction points for various taxa, some of which are reputed to carry zoonoses. As such, these burrows should be considered as potential “One Health hotspots” to be monitored, especially when frequented by primates in contact with rangers or tourists.

## Author Contributions

**Cédric Vermeulen:** conceptualization (lead), funding acquisition (lead), investigation (supporting), methodology (equal), supervision (lead), validation (equal), writing – original draft (lead), writing – review and editing (lead). **Jérôme Vandebos:** data curation (lead), investigation (equal), writing – review and editing (supporting). **Daelemans Virginie:** data curation (equal), investigation (equal), writing – review and editing (supporting). **Simon Lhoest:** validation (equal), writing – review and editing (equal).

## Ethics Statement

No animals were captured, handled, or disturbed during this study. The camera traps were installed to avoid disturbing the animals, and the observations are based on photos and videos taken in the absence of observers.

## Conflicts of Interest

The authors declare no conflicts of interest.

## Supporting information


Photo S1.

Photo S2.

Data S1.

## Data Availability

The complete database from which these preliminary observations are derived is not yet online, due to the heavy weight of photographic and film data and because the entire database is not necessary for what we establish in the paper. We have added a number of videos to this review to support our argument.
