# Supplementary figures and images for: Primates in Burrows: A Cause for Concern? Observations From a One Health Perspective at Niokolo Koba National Park, Senegal
Source: Ecol Evol. 2025 Apr 7;15(4):e71062. doi: 10.1002/ece3.71062 (PMC11974457; doi:10.1002/ece3.71062)

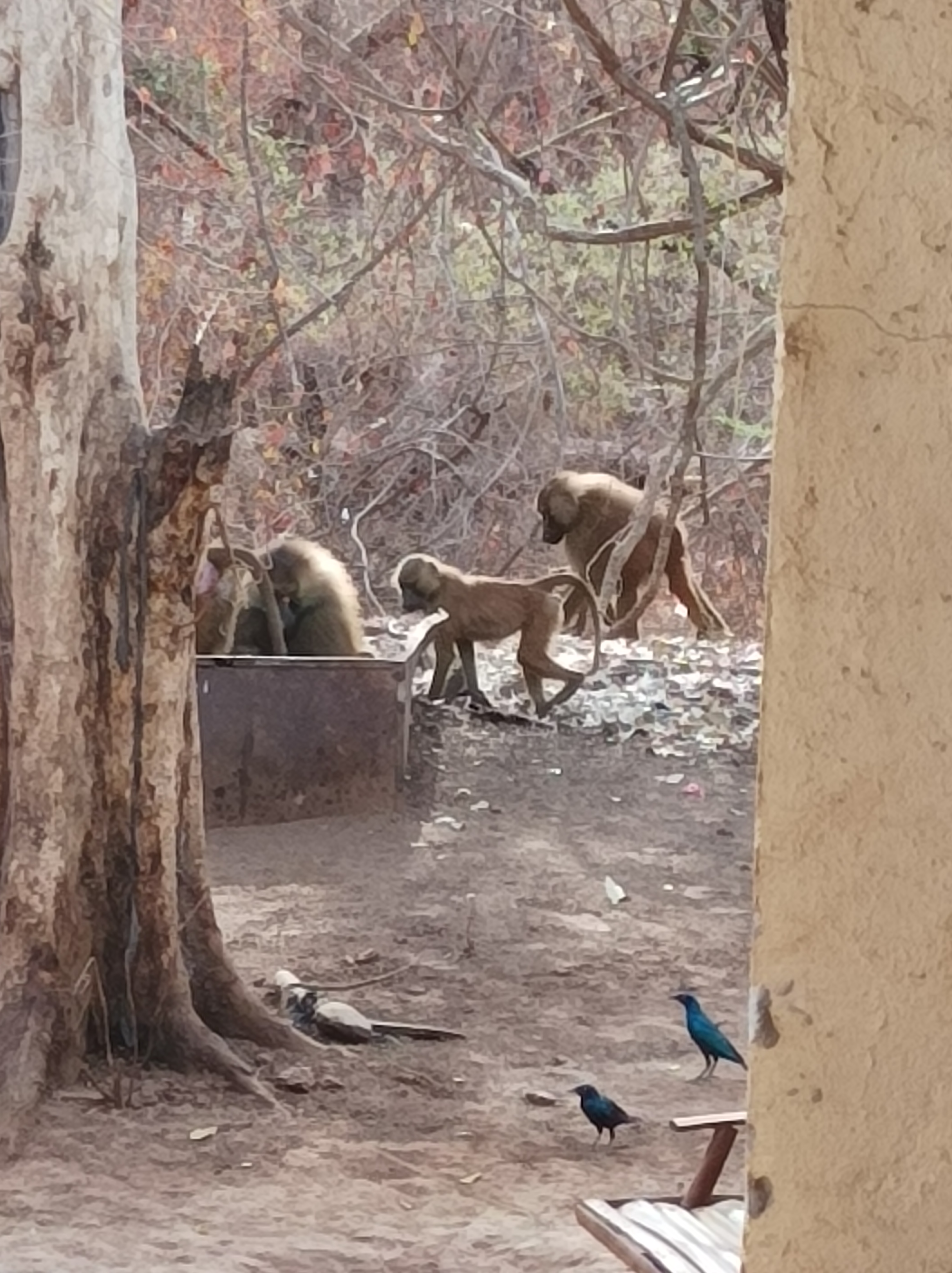

Supplement: Supplementary file 1 — Photo S1. [file ECE3-15-e71062-s002.jpg]

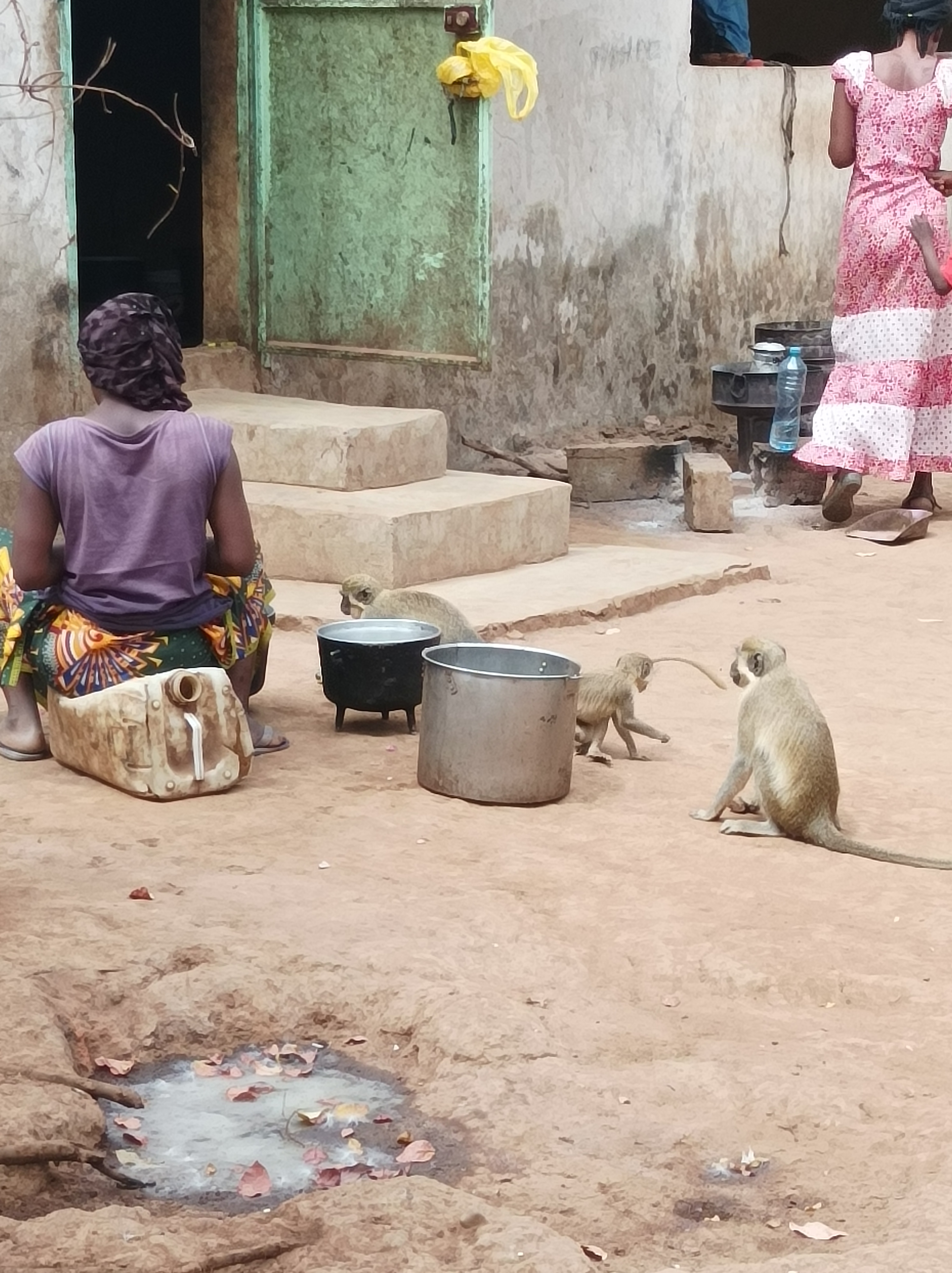

Supplement: Supplementary file 2 — Photo S2. [file ECE3-15-e71062-s001.jpg]
